# Size-selective glomerular filtration as a hallmark of premature kidney ageing in nondiabetic individuals

**DOI:** 10.1093/ckj/sfaf208

**Published:** 2025-07-02

**Authors:** Agne Laucyte-Cibulskiene, Christopher Nilsson, Amra Jujic, Hannes Holm, Anders Christensson

**Affiliations:** Department of Clinical Sciences, Lund University, Malmö, Sweden; Department of Nephrology, Skåne University Hospital, Malmö, Sweden; Department of Clinical Sciences, Lund University, Malmö, Sweden; Department of Nephrology, Skåne University Hospital, Malmö, Sweden; Department of Clinical Sciences, Lund University, Malmö, Sweden; Department of Cardiology, Skåne University Hospital, Malmö, Sweden; Lund University Diabetes Centre, Lund University, Malmö, Sweden; Department of Clinical Sciences, Lund University, Malmö, Sweden; Department of Cardiology, Skåne University Hospital, Malmö, Sweden; Department of Clinical Sciences, Lund University, Malmö, Sweden; Department of Nephrology, Skåne University Hospital, Malmö, Sweden

**Keywords:** advanced glycation end products, cystatin C, estimated glomerular filtration rate, kidney ageing, size-selective kidney filtration

## Abstract

**Background:**

During the last decade, evidence has emerged on selective glomerular hypofiltration syndromes (SGHS), defined by selectively reduced filtration of middle-sized molecules irrespective of kidney function and resulting in a low ratio between cystatin C– and creatinine-based estimated glomerular filtration rates (eGFRcys/eGFRcr). We aimed to examine whether SGHS is a hallmark of premature kidney ageing manifested by accumulation of advanced glycation end-products (AGEs) and endothelial dysfunction in a population free from chronic kidney disease and diabetes.

**Methods:**

A total of 3804 participants of The Malmö Offspring Study (MOS) underwent AGE (skin autofluorescence acquired), eGFRcys and eGFRcr measurements. AGEs ≥1.6 were categorized as high, and AGEs <1.6 as normal. Reactive hyperemia index (RHI) was available in 2204 participants; RHI <1.67 indicated endothelial dysfunction (ED), and RHI ≥1.67 normal endothelial function (EF). Combining AGEs and RHI, four groups were compared: (group 1) AGEs <1.6 and EF, (group 2) AGEs <1.6 and ED, (group 3) AGEs ≥1.6 and EF, and (group 4) AGEs ≥1.6 and ED.

**Results:**

Lower eGFRcys/eGFRcr ratio was associated with an increase in AGEs in men and women. After adjusting for cardiovascular factors, lower eGFRcys/eGFRcr ratio was associated with AGE accumulation in men older than 30 years. The ‘AGEs ≥1.6 and ED’ and ‘AGEs ≥1.6 and EF’ groups showed the highest prevalence of eGFRcys/eGFRcr under 0.9 (in men, 22% vs 19.7%, in women 19.5% vs 16%, respectively).

**Conclusions:**

Accumulation of AGEs resulting from SGHS leads to age dependent changes in glomerular basement membrane and increased selectivity for middle-sized molecules. Presence of these findings in younger individuals supports the hypothesis that SGHS is a model of early kidney ageing, occurring before decline in kidney function.

## Graphical Abstract

**Graphical Abstract:**
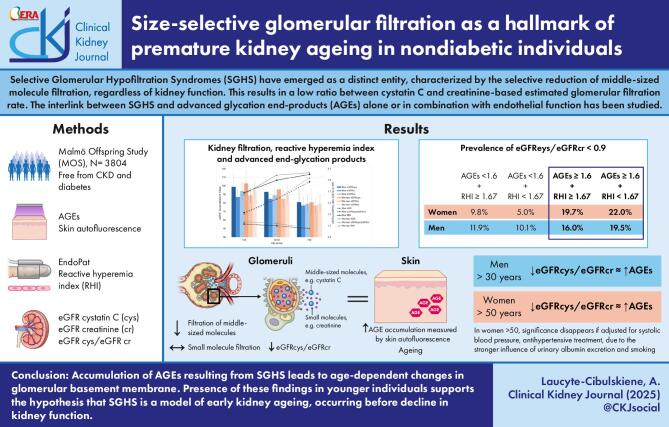

KEY LEARNING POINTS **What was known:**

- Cystatin C alone or in combination with creatinine increases precision in kidney function estimation.
- Selective glomerular hypofiltration syndromes (SGHS), defined by selectively reduced filtration of middle-sized molecules irrespectively of kidney function, is more prevalent among older adults and presents higher all-cause mortality and cardiovascular morbidity rates in the general population.

**This study adds:**

- This is the first study investigating SGHS as a model of accelerated kidney ageing in a sample selected from the general population that is free from diabetes and chronic kidney disease.
- Skin autofluorescence-derived accumulation of advanced glycation end-products (AGEs) secondary to SGHS leads to age-dependent and sex-specific changes in glomerular basement membrane and increased selectivity for middle-sized molecules, with pronounced effects in men above 30 years and women above 50 years of age.
- Individuals with endothelial dysfunction and higher levels of AGEs defined a group of individuals with the highest prevalence of SGHS.

**Potential impact:**

- Our work proposes predominant mechanisms behind mutual AGEs and SGHS roles in early kidney ageing and explains previously reported lower prevalence of SGHS among younger individuals.
- Our findings suggest that the value of adding reactive hyperemia index for understanding cardiovascular and kidney interaction is controversial, possibly affected by vascular tone in younger individuals and different timing in cardiovascular and kidney ageing.

## INTRODUCTION

Organ ageing refers to the gradual structural and functional loss in the human body. Natural kidney ageing is characterized by diverse pathophysiological mechanisms also observed in chronic kidney disease (CKD) [1]. The microscopic structural changes include microvascular rarefaction, interstitial and pericapsular fibrosis, arteriosclerosis, glomerulosclerosis, thickening of glomerular basement membrane, tubular atrophy and tubular distinction, and other related alterations [2]. CKD is a model of premature kidney ageing that deviates from the expected ageing trajectory and thus, increases the gap between biological and chronological age [3].

The connection between vascular and kidney ageing is well described [3]. The cause-and-effect reasoning, however, in this relationship is debatable. Emerging evidence shows that changes in vasculature and kidneys might coincide and have bidirectional influence [4]. This cardiovascular and kidney interaction in the early stages of kidney ageing, both biological and premature, is not well defined [5].

Age-related kidney functional loss can be indirectly estimated by glomerular filtration rate (eGFR). Some reports propose that a kidney age-chronological age score can help overcome pitfalls and failures of creatinine-based estimated GFR in older adults [6]. Cystatin C (cys) alone or in combination with creatinine increases precision in kidney function estimation and is recommended by Kidney Disease: Improving Global Outcome (KDIGO) guidelines [7, 8]. During the last decade, evidence for selective glomerular hypofiltration syndromes (SGHS) has emerged, marked by both shrinkage and elongation of glomerular pores, and defined by selectively reduced filtration of middle-sized molecules (10–30 kDa) compared with smaller ones (<1 kDa), irrespectively of kidney function [9]. SGHS is observed regardless of the presence of non-renal factors that may influence cystatin C and creatinine concentrations, such as creatinine's correlation with muscle mass and dietary protein intake from cooked meat, and cystatin C's correlation with fat mass, and its potential impact from glucocorticoid treatment and thyroid disorders [8, 10, 11]. SGHS is usually measured as a ratio between eGFRcys and eGFRcr or the difference in eGFR by creatinine versus cystatin C [8, 12–14]. A common definition of SGHS is eGFRcys/eGFRcr <0.7, although this threshold depends on the methods used for measuring cystatin C and creatinine [13]. Glomerular filtration barrier is mainly composed of glomerular basement membrane (GBM), fenestrated endothelial cells (ECs) and podocytes. Restricted filtration of middle-sized molecules compared with small molecules like creatinine may be due to shrinking of the pores (shrunken pore syndrome) or elongation of the pores (elongated pore syndrome) [15]. Glomerular ECs and thickening of GBM may be a key pathological pattern in SGHS. The knowledge on how glomerular ECs communicate with cardiovascular ECs is limited. Single-cell omic studies defined similar ageing-related EC phenotype kidney and heart manifested with increased expression of senescent cell signatures [16]. Hence, endothelial dysfunction may contribute to selective glomerular hypofiltration.

SGHS is related to higher all-cause mortality, cardiorenal syndrome, pregnancy-related hypertension and atherosclerosis [17–20]. To date, a single study has proposed that adequate dietary protein intake may be a potential treatment option for mitigating the risks associated with SGHS [21]. What role SGHS plays in vascular ageing is still under investigation. In middle-aged and elderly individuals from the Swedish urban population, SGHS failed to show any association with aortic stiffness [22]. Considering that cardiovascular and kidney ageing deviate from each other, SGHS most likely reflects premature kidney ageing [14, 23].

Advanced glycation end products (AGEs) are exogenously and endogenously non-enzymatically produced from sugars, proteins or lipids, and can be non-invasively measured using skin autofluorescence (SAF), a technique that captures the fluorescent signals emitted by AGEs in the skin [24]. They have pro-inflammatory and pro-oxidative properties and accumulate not only in hyperglycemic states, but also in CKD and accelerate vascular ageing. Notably, AGEs have hepatic and kidney clearance, and these organs maintain AGEs homeostasis. In animal models, AGEs mitigated vasodilation and endothelial function by altering nitric oxide and endothelial nitric oxide synthase expression in endothelial cells in case of diabetes [25–28]. The endothelial dysfunction due to excess of circulating AGEs in nondiabetic patients with CKD stages 2, 3 and 5 has also been confirmed [29]. Endothelial dysfunction can be detected through the measurement of flow-mediated dilation and represents the early stages of vascular remodelling, further progressing to cardiovascular diseases (CVD). SGHS, like AGEs and endothelial function, has been related to atherosclerosis, CKD, heart failure, frailty and all-cause mortality [12, 13, 17–19, 30]. With this work we sought to enlighten the interlink between AGEs, endothelial function and size-selective kidney filtration in individuals without pronounced CKD and diabetes that might be a key to understanding cardiovascular and kidney interaction.

## MATERIALS AND METHODS

### Participant selection

Participants of the Malmö Offspring Study (MOS) [31] were enrolled. MOS is a population-based cohort study (*n* = 5277, age 18–71 years) that includes descendants—adult children (generation 2) and grandchildren (generation 3)—of the Malmö Diet and Cancer Study. MOS started in 2013 and was completed in 2021. The primary objective of MOS was to plot family clustering for epigenetic features of cancer, diabetes, CVD and dementia. We selected individuals who met the following inclusion criteria:

- Average eGFR (the mean of creatinine- and cystatin C–based eGFR) ≥60 mL/min/1.73 m^2^
- No known diabetes mellitus and fasting glycaemia <7.0 mmol/L
- Present data on SAF

**Figure 1:**
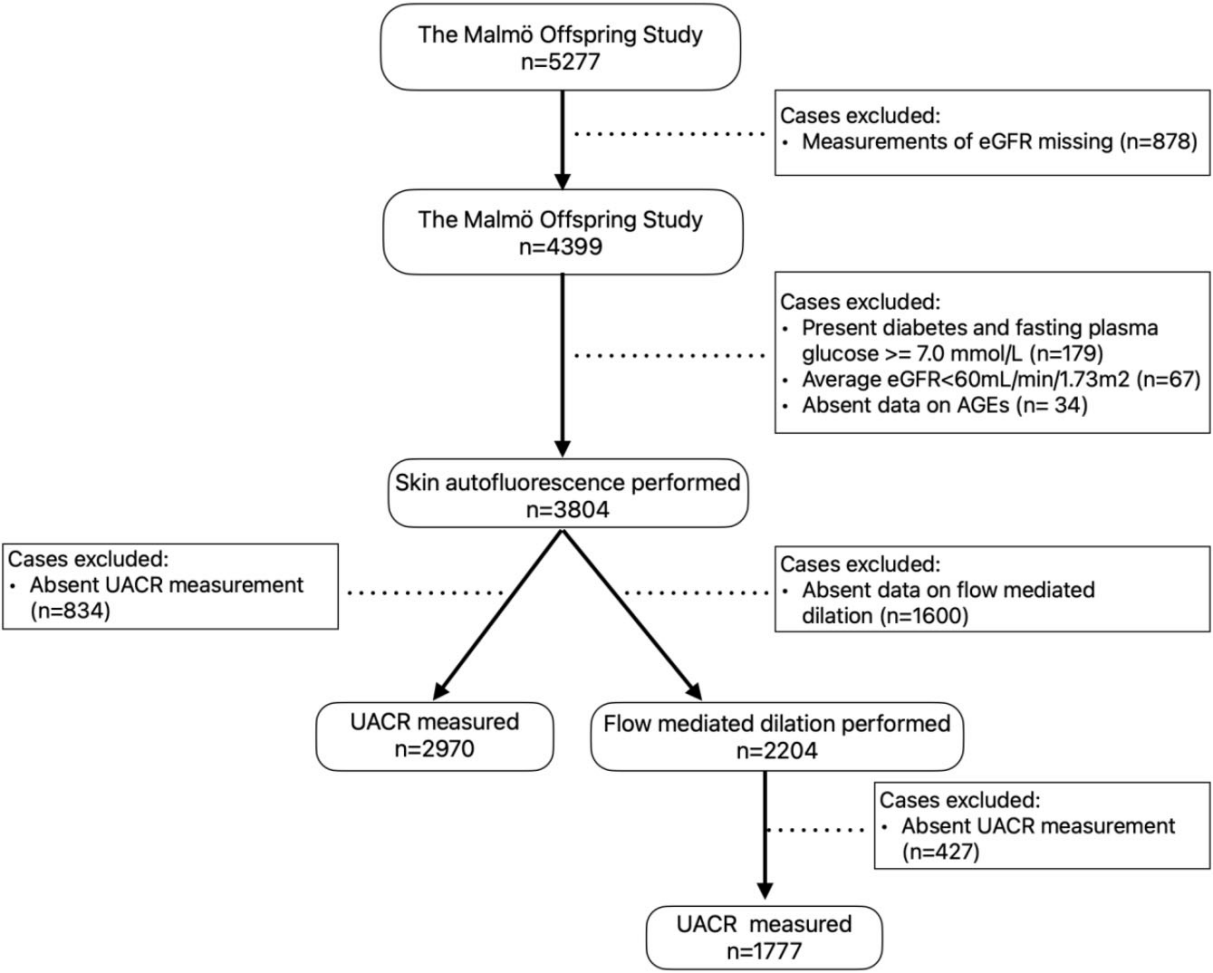
Flowchart of selected individuals.

A total of 3804 subjects underwent SAF alongside eGFR measurements. Of these subjects, 78% (*n* = 2970) had additional data on urine albumin-to-creatinine ratio (UACR), while 58% (*n* = 2204) underwent evaluation of endothelial function. Forty seven percent (*n* = 1777) had both endothelial readings and UACR measurements (Fig. 1).

All participants had given written informed consent, and the study was carried out in line with the ethical principles of the Declaration of Helsinki. The ethical approval was obtained from Swedish ethical authorities (Dnr. 2012/594).

### Demographics, biochemistry and blood pressure

Data sources and assessment methods are listed in Supplementary data, Table S1.

### Skin autofluorescence of advanced glycation end products

At room temperature, the skin was illuminated by 300–420 nm ultraviolet light. The spectrometer registered emitted light of skin. Three readings on individuals’ non-dominant arm were recorded, and the average value in arbitrary units (AU) was calculated. SAF was utilized to measure AGEs (AGEs Reader, DiagnOptics, Groningen, The Netherlands) [31]. Mean AGE levels in MOS cohort were 1.6, therefore AGEs ≥1.6 were considered as high, AGEs <1.6 as normal.

### Endothelial function

Flow-mediated dilation was used to measure endothelial function (EndoPat, Itamar, Israel). After 5 min of resting, the cuff on the non-dominant upper arm was inflated to at least 200 mmHg (or 60 mmHg above systolic pressure), and after 5 min, occlusion was released and the signal amplitude for another 5 min registered [31]. A ratio between post- and pre-occlusion signals is then calculated and presented as the reactive hyperemia index (RHI).

The established cut-off value below 1.67 for RHI, as proposed by Tanaka *et al*., was used in defining endothelial dysfunction (ED) [32]. RHI <1.67 indicated ED, and RHI ≥1.67 normal endothelial function (EF) [32]. The phenomenon of pseudo-ED in individuals under 30 years old was also addressed by cautious interpretation of low RHI values. In this subpopulation, EndoPat acquired RHI can be affected by measurement related errors and inability of healthy arteries to dilate post-occlusion.

### Kidney function

Plasma cystatin C and creatinine-based estimation of glomerular filtration was performed according to: (i) cystatin C–based (eGFRcys) Caucasian, Asian, paediatric and adult cohort equation (CAPA) (eGFRcys); or (ii) the Lund-Malmö revised creatinine-based eGFR equation (LMrev) (eGFRcr) [33, 34]. The selection of eGFR equations was guided by previous work demonstrating the validity and comparable performance of eGFRcys equations, as well as the superiority of creatinine-based formulas such as LMrev and the European Kidney Function Consortium (EKFC) over the Chronic Kidney Disease Epidemiology Collaboration (CKD-EPI) equation in the Swedish population [35]. Selective glomerular hypofiltration syndromes were defined as a ratio between eGFRcys and eGFRcr. The average eGFR was determined as the mean of eGFRcys and eGFRcr. Albumin excretion was measured as UACR in spot urine from morning samples.

### Statistical analysis

Normally distributed continuous data are presented as means and standard deviations, and skewed data as medians and interquartile ranges. Percentages and the number of cases in the brackets were used for categorical variables. When appropriate, means were compared with Student’s *t*-test (two categories) and analysis of variance (ANOVA) analysis (two or more categories). Chi-squared tests were used to evaluate the relationships between categorical variables.

Pearson correlation heatmap was used to illustrate the strength of positive (red colour) and negative (blue colour) associations among variables, with a focus on AGEs, RHI (natural logarithm), eGFRcys, eGFRcr, eGFRcys/eGFRcr and UACR (natural logarithm).

Women and men were analysed separately due to known biologic differences in kidney function and ageing. Sex-disaggregated outputs were presented.

To investigate whether the relationship between kidney function estimates and AGE level is age-dependent, an interaction analysis was conducted. First, centred variables were calculated for age, eGFR average, eGFRcys, eGFRcr and eGFRcys/eGFRcr by subtracting the mean value from each raw variable value and dividing by the standard deviation. Next, interaction variables were constructed by multiplying the centred age values with the centred values of each kidney function marker, such as ‘age (centred) * eGFR average (centred)’, ‘age (centred) * eGFRcys (centred)’, ‘age (centred) * eGFRcr (centred)’ and ‘age (centred) * eGFRcys/eGFRcr (centred)’. Linear regression models were then employed, with AGE as the dependent variable and independent factors consisting of centred age, centred kidney function markers (eGFR average, eGFRcys, eGFRcr or eGFRcys/eGFRcr), and the interaction between age and kidney function estimates. Due to the relatively small effect size, the subjects were divided into age groups as follows: <30 years (548 men and 568 women); 30–50 years (682 men and 673 women) and >50 years (649 men and 684 women).

AGE was the dependent variable in linear regression models. The unadjusted and nested linear models for exploring the relationship between AGEs and eGFRcys/eGFRcr ratio (independent variable) were utilized, and regression coefficients and their 95% confidence intervals were presented as forest plots. Model 1 represented eGFRcys/eGFRcr adjusted for eGFRaverage; Model 2—Model 1 additionally adjusted for age and body mass index (BMI); Model 3—Model 2 additionally adjusted for smoking, fasting plasma glucose, triglycerides, low-density lipoprotein cholesterol (LDL)-cholesterol, high-density lipoprotein cholesterol (HDL)-cholesterol and HbA1c; and Model 4—Model 3 additionally adjusted for systolic pressure, antihypertensive treatment and log(UACR). To mitigate the risk of multicollinearity, we employed the Variation Inflation Factor (VIF) to assess the correlation between variables. A VIF value <2.0 was considered acceptable, indicating minimal multicollinearity. Notably, the eGFRcys/eGFRcr ratio did not exhibit significant intercorrelation with average eGFR in Models 1–4, as the VIF values remained <2.0. However, age showed borderline multicollinearity with other variables in Models 1–3 (VIFs of 2.1 in men and 1.9 in women) and in Model 4 (VIFs of 2.3 in men and 2.2 in women).

The MOS cohort initially aimed to measure RHI in all participants. However, challenges in evaluating EF in adults under 30 years (error values, artefacts) contributed to small sample size in this group (59 men and 82 women). In contrast, in age group 30–50 years, 396 men and 398 women had EF measurements, and in age group >50 years, 617 men and 656 women. Four different categories were defined by combining AGE values and RHI: (group 1) AGEs <1.6 and EF, (group 2) AGEs <1.6 and ED, (group 3) AGEs ≥1.6 and EF, and (group 4) AGEs ≥1.6 and ED. One-way ANOVA and analysis of covariance tests were used to compare parameters between sexes and among these categories.

## RESULTS

### Study participants

Participant characteristics are presented in Table 1. Women had smaller waists (*P* = .006) and lower BMI (*P* = .035), as expected. Higher HDL-cholesterol but lower LDL-cholesterol levels were observed among women (*P* < .001 and *P* = .013). Men had lower eGFRcys but similar eGFRcr. eGFRaverage and eGFRcys/eGFRcr were significantly higher in women (*P* < .001). Systolic blood pressure (SBP) was slightly higher but within the normal range in men (*P* < .001). Sixty-one participants had UACR above 3 mg/mmoL (32 men and 29 women), in the range from 3.1–245 mg/mmoL.

**Table 1:** Descriptives of individuals enrolled in the analysis.

	Women	Men	
	*N* = 1925	*N* = 1879	*P*-values
Age, years	42 (15)	42 (15)	.921
Waist, cm	89 (14)	90 (13)	.006
BMI, kg/m^2^	25.8 (4.7)	26.1 (4.7)	.035
Hypertension	18 (337)	18 (346)	.353
Antihypertensive treatment	8 (159)	9 (170)	.324
History of CVD	2 (38)	2 (39)	.769
Smoking	14 (277)	14 (263)	.309
Biochemistry			
Fasting glycaemia	5.2 (0.6)	5.2 (0.6)	.669
TG, mmoL/L	0.9 (0.7)	0.9 (0.8)	.541
HDL, mmoL/L	1.64 (0.48)	1.59 (0.47)	<.001
LDL, mmoL/L	3.14 (0.94)	3.22 (0.96)	.013
HbA1c, IFCC units	34 (4)	34 (4)	.249
Kidney function			
eGFRcys, mL/min/1.73 m^2^	93 (20)	91 (18)	<.001
eGFRcr, mL/min/1.73 m^2^	83 (11)	83 (11)	.238
eGFRaverage, mL/min/1.73 m^2^	88 (13)	87 (12)	<.001
eGFRcys/eGFRcr	1.13 (0.25)	1.10 (0.22)	<.001
Ln(UACR)^a^	–0.94 (1.0)	–1.0 (0.95)	.271
Vascular parameters			
SBP, mmHg	115 (15)	116 (15)	.001
DBP, mmHg	72 (10)	71 (10)	.222
HR, bpm	63 (10)	63 (10)	.404
SAF			
AGE (AU)	1.6 (0.5)	1.6 (0.6)	.271
Flow-mediated dilation			
RHI	2.1 (0.8)	2.1 (0.8)	.912

Normally distributed continuous variables presented as mean (± standard deviation), skewed variables; ^a^median (interquartile range), categorical variables as % (number).

UACR is in mg/mmoL.

TG, triglycerides; HbA1c, glycolized haemoglobin; HR, heart rate; AU, arbitraty units.

### Association of advanced glycation end products with selective glomerular hypofiltration syndromes

**Figure 2:**
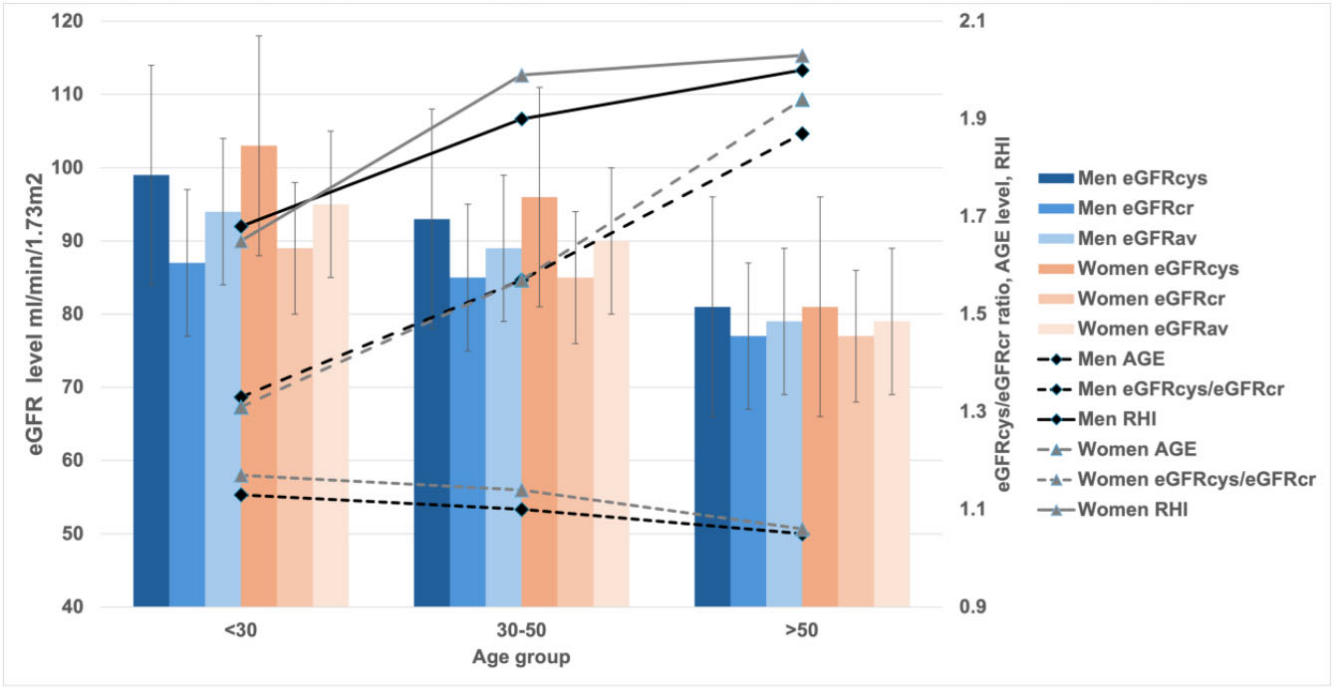
Age and sex divided distribution of AGEs, RHI and kidney function.

The interaction analysis revealed that in men, neither the average eGFR nor the eGFRcys showed an age-dependent relationship with AGEs (Supplementary data, Table S2). In contrast, in women, the same results were observed for eGFR average, but lower eGFRcys was associated with higher AGE levels. Higher eGFRcr had an age-dependent relationship with AGEs in both sexes. Notably, lower eGFRcys/eGFRcr was related to higher AGE levels with increasing age in both men and women. It is worth noting that the effect sizes of these interaction analyses were relatively small (Supplementary data, Table S2). However, when the age groups were analysed (Supplementary data, Table S3), the effect size increased, revealing a more pronounced negative association between eGFRcys/eGFRcr and AGEs in men older than 30 years. In women, the relationship was positive in ages under 50 years, but became negative only in individuals older than 50 years. The distribution of AGEs, RHI, and kidney function by age and sex is depicted in Fig. 2.

**Figure 3:**
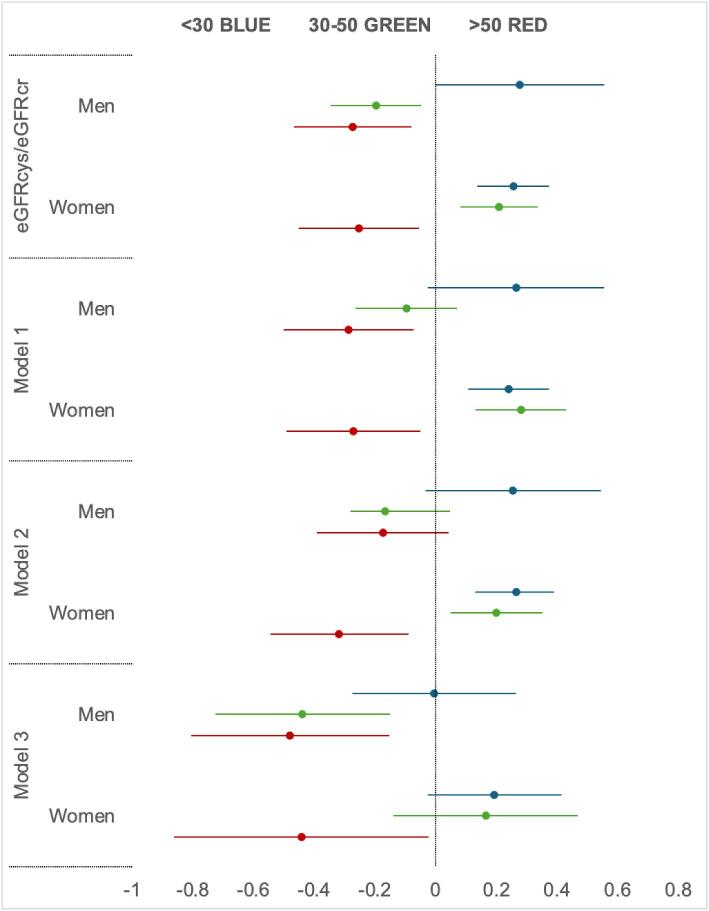
Linear regression coefficients for models where AGEs are a dependent factor. Forrest plot diagram.

Correlation coefficients are presented in Supplementary data, Fig. S1. Age- and sex-specific linear regression analysis showed a significant inverse relationship between eGFRcys/eGFRcr and AGEs in men aged 30–50 and >50 years (Models 1–3 depicted in Fig. 3, and Model 4 in Supplementary data, Fig. S2). Smoking was associated with higher AGE levels in the age group <30 years [beta 0.207, 95% confidence interval (CI) (0.135;0.278)] and >50 years [beta 0.202, 95% CI (0.131;0.274)].

In women older than 50 years, the eGFRcys/eGFRcr ratio and AGEs association was attenuated after additional adjustment for SBP, antihypertensive treatment and UACR (Model 4, Supplementary data, Fig. S2). In this group, smoking [beta 0.190, 95% CI (0.111;0.269)] and UACR [beta 0.068, 95% CI (0.018;0.117)] were associated with AGEs. The other significant factors associated with AGE levels were: in the age group under 30 years—triglycerides [beta 0.147, 95% CI (0.072;0.223)] and UACR [beta 0.007 95% CI (0.014; 0.078)]; in the age group 30–50 years—age [beta 0.016, 95% CI (0.008;0.024)], smoking [beta 0.097, 95% CI (0.034;0.160)], HDL-cholesterol [beta 0.231, 95% CI (0.012;0.350)] and LDL-cholesterol [beta –0.075, 95% CI (–0.136;–0.015)]. Interestingly, a trend towards a positive relationship between eGFRcys/eGFRcr and AGEs was observed in women younger than 30 years.

### Interaction between advanced glycation end products, endothelial function and size-selective glomerular filtration

The crosstalk between AGEs and endothelial function and its possible relationship to parameters of kidney function were evaluated in four groups (see definitions in Statistical analysis). As presented in Supplementary data, Table S4, the youngest individuals, with the lowest previous CVD and hypertension burden, were attributed to group 2. Men in this group had similar metabolic profile (glycaemia, HbA1c, lipids, waist circumference and BMI) to group 1 and group 3. In women from group 2, glycaemia, HbA1c and LDL-cholesterol were significantly lower than in group 3 (*P* = .049, *P* = .014 and *P* = .003, respectively). Waist circumference (*P* = .009) and BMI (*P* = .014) showed the same trend. eGFRcys and eGFRcys/eGFRcr ratio in group 2 in both sexes were the highest compared with other groups (*P* < .001), and log(UACR) in men was lower than in group 3 (*P* = .031) and group 4 (*P* = .011). The increased RHI in group 2 represents pseudo-ED.

eGFRcr was evenly distributed across the groups. Indeed, groups 3 and 4 in women shared most of the characteristics, e.g. eGFRcys, eGFRcr, eGFRcys/eGFRcr ratio, eGFRaverage, ln(UACR), etc., except from SBP (*P* = .009) and diastolic blood pressure (DBP) (*P* = .008). In men, additional discrepancy in triglyceride level (*P* = .005) was observed.

**Figure 4:**
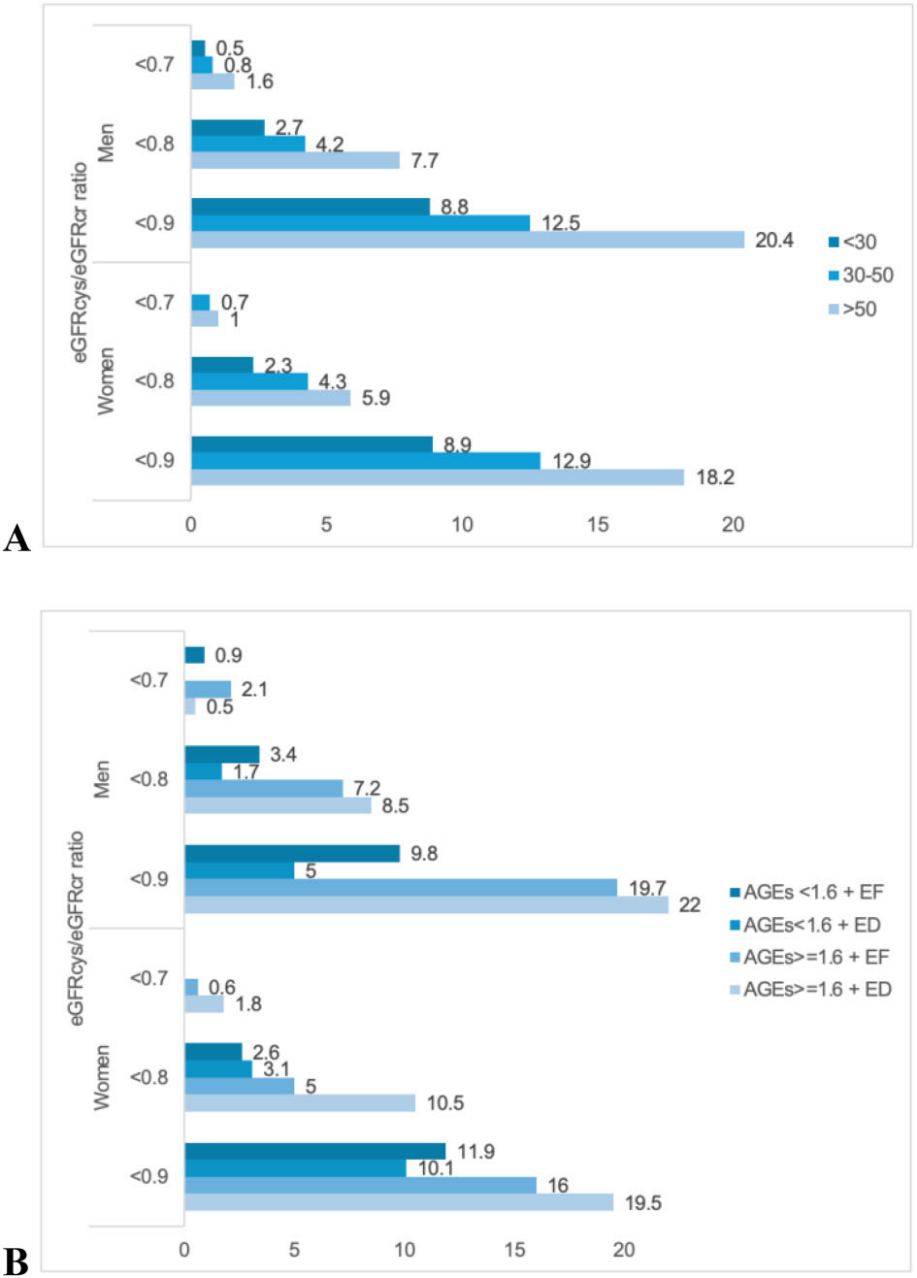
Prevalence of SGHS. (**A**) Prevalence of different eGFRcys/eGFRcr cutoffs among age groups: under 30, 30 to 50, and over 50. (**B**) Prevalence of different eGFRcys/eGFRcr cutoffs according to AGE levels in combination with endothelial function.

We then investigated the prevalence of selective glomerular hypofiltration syndrome defined with cut-offs 0.7, 0.8 and 0.9 for eGFRcys/eGFRcr ratio (Fig. 4A and B). SGHS prevalence was age-dependent (Fig. 4A) and slightly higher in men. When analysing diverse pathophysiological patterns, SGHS was primarily prevalent in men attributed to group 3 (AGEs ≥1.6 + EF) and group 4 (AGEs ≥1.6 + ED) if using a cut-off of 0.8 and 0.9. In women, the same trend was noticed but to a lesser extent. The lowest prevalence of SGHS was in group 2 (AGEs <1.6 + ED) in both sexes (Fig. 4B).

## DISCUSSION

This study supports the hypothesis that AGE accumulation measured by SAF alone or in combination with endothelial function uncovers SGHS in individuals without advanced CKD and diabetes. Both men and women had a decline in eGFRcys/eGFRcr followed by an increase in AGEs, which progressed with age and more rapidly in women. After adjustment for cardiovascular factors, a lower eGFRcys/eGFRcr ratio was associated with higher AGE levels in men older than 30 years. In women older than 50 years, this interaction was attenuated and was mainly influenced by smoking and urinary albumin excretion. Endothelial dysfunction and higher AGEs defined a group of individuals with the highest prevalence of SGHS. Conversely, individuals with endothelial pseudo-dysfunction and normal values of AGEs had the highest eGFRcys/eGFRcr ratio, mainly explained by the higher eGFRcys levels.

**Figure 5:**
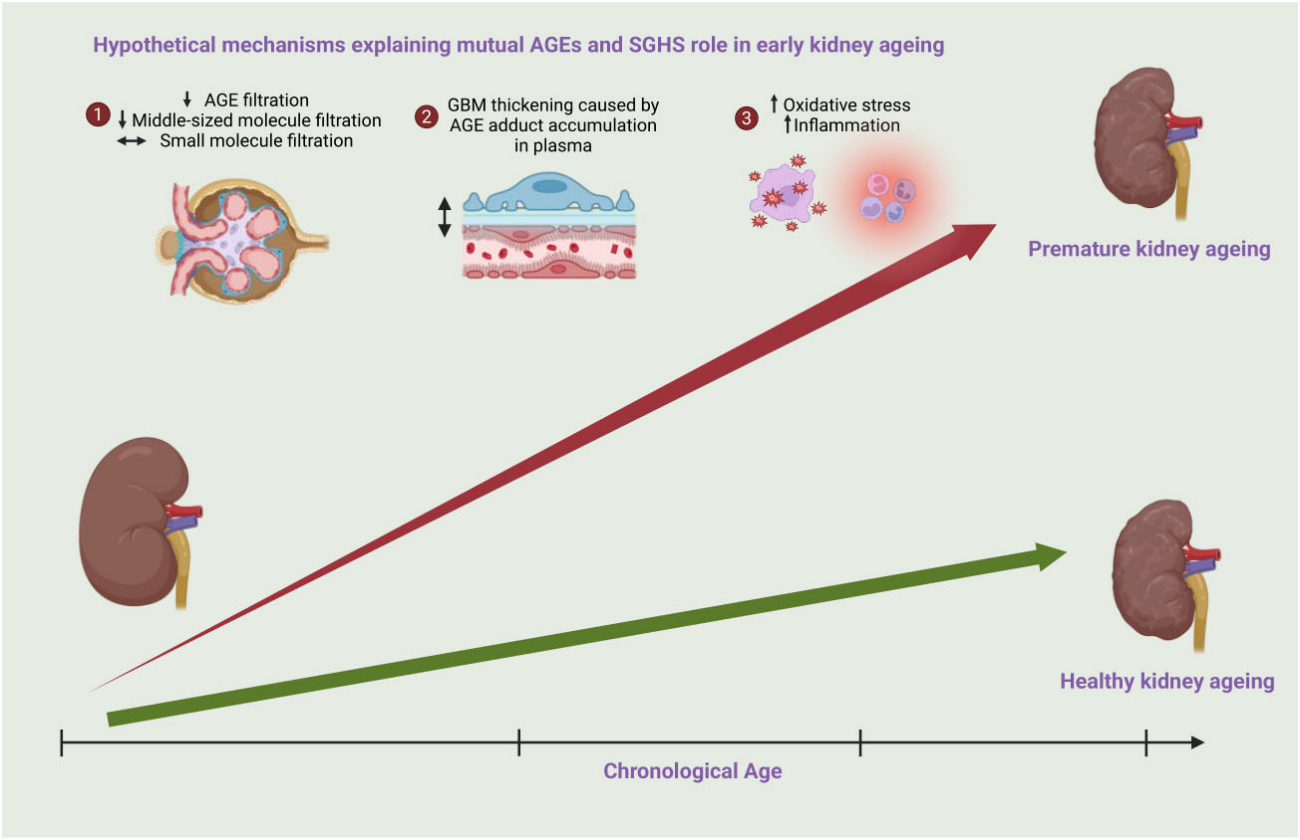
Hypothetical mechanisms explaining mutual AGEs and SGHS roles in early kidney ageing.

Vascular ageing is a continuous and progressive process involving several structural changes. SGHS depicts changes in glomerular filtration of middle-sized molecules most likely due to structural changes in the endothelium and membrane. Therefore, we hypothesize that SGHS may be a marker of premature kidney ageing. However, different methods to describe the early vascular changes may give different results concerning SGHS due to what mechanism is most predominant. This study gives further insights and strengthens this hypothesis in several ways, as highlighted in Fig. 5 and further in the text: (i) altered filtration of AGEs through glomerular filtration barrier; (ii) glomerular basement membrane thickening caused by AGE accumulation; and (iii) upregulation of oxidative stress and inflammation. In any case, the relationship between SGHS and vascular function, i.e. endothelial function, as measured by RHI, was not apparent.

Several types of AGEs are dietary and endogenously produced, and only glucose- and fructose-derived AGEs are fluorescent and have molecular weight under 12 kDa [24, 36]. These AGE-free adducts—remnants after cellular proteolysis—are filtered through the glomerular filtration barrier and are particularly reabsorbed in the tubule. Their clearance is, however, lower than that of creatinine's and further declines with advancing CKD [24]. Interestingly, cystatin C is a 13 400 Da protein, with a molecular weight close to that of glucose- and fructose-derived AGEs [36, 37]. In contrast, creatinine is a 113 Da molecule, more than 100 times smaller that cystatin C and fluorescent AGEs [37]. Therefore, a possible explanation for our findings of an age-dependent increase in AGEs alongside the increased prevalence of SGHS is that age-related shrinking and elongation of pores in the glomeruli leads to the accumulation of fluorescent AGEs and cystatin C [37]. A comprehensive meta-analysis comparing the prevalence of SGHS across different age groups is currently lacking, making it difficult to directly compare our findings with existing literature. A review article by Grubb reported a prevalence range of 0.2%–36% across various cohorts, and recent data suggest a significant increase to approximately 75% in SGHS prevalence within 4 weeks after heart transplantation [37, 38].

The relationship between SGHS and endothelial function was less clear, despite the fact that 31% of the 2204 individuals analysed in this study had endothelial dysfunction, as defined by a RHI >1.67. Moreover, individuals with SAF measured AGE levels >1.6 had a higher prevalence of SGHS and a worse metabolic profile. The presence of low RHI in individuals with AGE levels <1.6 is controversial, suggesting pseudo-endothelial dysfunction and is associated with the lowest prevalence of SGHS [39]. This contradiction in RHI values was previously described in children and could be due to lower peripheral vascular resistance dependent on sympathetic nerve activity [40, 41]. Although sympathetic overactivity is well-defined in CKD another possible explanation could be the protective effect of cystatin C on endothelial cells shown in animal models [42, 43]. Whether individuals in low RHI and low AGE groups have age-independent lower risk for cardiovascular or kidney outcomes should be researched further. This selected cohort is young and in good health, and the prevalence of CV events is so far very low.

Our finding that men older than 30 years with SGHS had higher AGE levels, whereas this relationship disappeared in women above 50 years, may be attributed to different trajectories of age-related glomerular basement membrane (GBM) thickening and the later debut of vascular diseases in women. It might also be a result of AGE accumulation in GBM, and AGE-RAGE (receptor for AGEs) interaction stimulated synthesis of fibronectin and collagen type I, II and IV [44]. Moreover, GBM thickness has been attributed to SGHS in diabetic kidney disease, i.e. the thicker GBM the lower ratio of eGFRcys/eGFRcr, as shown by Öberg *et al*. [15]. So far, no other studies have explored possible histological changes in nondiabetic individuals with SGHS, though such studies are warranted. Previous knowledge that GBM thickening is a histological hallmark of age-related kidney structural changes suggests that SGHS might be a marker of premature kidney ageing [2]. Additionally, AGEs have been identified as a key factor contributing to kidney function decline in both natural and advanced aging in animal models, and SGHS could be one of the additional pathways by which AGEs precede CKD [45].

The other explanation for how AGEs and SGHS interact is the upregulation of oxidative stress and inflammation [46]. All kidney cells express RAGE which is upregulated in pathological conditions, e.g. CKD, diabetes mellitus, CVDs, cancer, atherosclerosis, infertility, gestational diabetes, Alzheimer's disease, etc. [47, 48]. When AGEs bind to RAGE, the oxidative stress and nuclear factor-kappa B (NF-κB) signalling, which is involved in inflammation and immunity, are activated [49]. This imbalance causes telomere attrition and cellular senescence, substantially causing age-related kidney structural and functional changes [50]. The same processes are responsible for cardiovascular ageing, though the trajectories of cardiovascular and kidney ageing can deviate from each other [23, 51]. Several studies in CKD stages 2–5 showed that higher levels of SAF-acquired AGEs unmasked subclinical atherosclerosis and predicted cardiovascular events and all-cause mortality [52–54]. Our findings, however, could not directly link AGEs with endothelial function, possibly due to the inclusion of participants from the general population without overt CKD. These individuals had significantly lower AGEs compared with the reference values published by Dutch researchers and those reported in the general population [55–58]. Despite that, we were able to determine the association between accumulation of AGEs with the lower eGFRcys/eGFRcr ratio independently on kidney function but reliant on age, supporting the hypothesis that SGHS is a model of early kidney aging before the decline of glomerular filtration.

The cross-sectional design is one of the limitations of this work, therefore, causal relationships could not be identified. Additionally, statistical models were not adjusted for non-renal factors that might influence the eGFRcys/eGFRcr ratio. The selection of individuals with a low CV risk burden leads to a lower prevalence of CV events later in life. Other potential outcomes, e.g. incident kidney disease, prescription of medications should be considered in further analysis of AGE accumulation and endothelial function.

No other study has investigated SGHS as a model of accelerated kidney ageing. This novel approach sheds light on the complexity of natural and pathological ageing in the human body. We have previously published pioneering data on the relationship between vascular ageing and SGHS and will continue to explore this field [22].

To conclude, SAF indirectly reflects the selective filtration of molecules in the kidneys, even in the absence of diabetes and advanced CKD. In women older than 50 years, this observed pattern disappears due to the more decisive influence of urinary albumin excretion and smoking on AGE levels. Accumulation of AGEs resulting from SGHS leads to age-dependent changes in glomerular basement membrane and increased selectivity for middle-sized molecules. Presence of these findings in younger age supports the hypothesis that SGHS is a model of early kidney ageing before the glomerular filtration starts to decline. The addition of RHI for understanding cardiovascular and kidney interaction is controversial, possibly affected by vascular tone in younger individuals and different timing in cardiovascular and kidney ageing. Future research should focus on other indices, such as the flow-mediated dilation index, for a better understanding of cardiovascular and kidney interactions.

## Supplementary Material

sfaf208_Supplemental_File

## Data Availability

The application to the Chair of the Steering Committee for the Malmö cohorts can be sent for data extraction used in this study. Sample and data extraction fees and additional information are available on a website: https://www.malmo-kohorter.lu.se/english.
