# Supplementary material for: Size-selective glomerular filtration as a hallmark of premature kidney ageing in nondiabetic individuals
Source: Clin Kidney J. 2025 Jul 2;18(8):sfaf208. doi: 10.1093/ckj/sfaf208 (PMC12451684; doi:10.1093/ckj/sfaf208)
Supplement: sfaf208_Supplemental_File [file sfaf208_Supplemental_File.pdf]

**Supplemental Table S1. Description of data sources and study variables available in The Malmö Offspring Study dataset.**

| Measurement | Variable | Assessment method/Data source |
|---|---|---|
| Socio-demographic data | age, sex, history of CVD | Questionnaire |
| Anthropometric data | Weight (kg), height (cm), waist circumference (cm), body mass index (kg/m2) | Measurements were performed using calibrated scales and measurement tapes; BMI expressed as weight(kg)/height(m)$^2$ |
| Smoking status | Current/former smoking and use of nicotine-patches | Questionnaire, self-reported |
| Diabetes | Prevalent diabetes | Self-reported diagnosis of type 2 diabetes, or diagnosis of diabetes at The Swedish National Patient Register, or the use of antidiabetic medication recorded in Swedish Prescribed Drug Register, or fasting plasma glucose ≥7 mmol/L on two separate occasions. |
| Hypertension | Prevalent hypertension | Diagnosis registered in the Swedish National Prescribed Drug Register, or systolic blood pressure >140 mmHg, diastolic blood pressure >90mmHg, or antihypertensive treatment. |
| Medication | Use of antihypertensive, lipid-lowering and antidiabetic medication. | Collected from the Swedish National Prescribed Drug Register using ATC-codes. |
| Blood work | lipid status (triglycerides (TG), high-density lipoproteins (HDL), low-density lipoproteins (LDL)), glycemia, glycated hemoglobin (HbA1c), creatinine and cystatin C. | Collected after 10 hours of overnight fasting; carried out at the Department of Clinical Chemistry, Skåne University Hospital, Malmö, Sweden. |
| Urine sample | Urine albumin-to creatinine ratio (UACR) | Spot urine from morning samples used; carried out at the Department of Clinical Chemistry, Skåne University Hospital, Malmö, Sweden. |
| Blood pressure | Systolic blood pressure (SBP), diastolic blood pressure (DBP). | An average value from two pulse wave analysis readings performed with applanation tonometry; Sphygmocor, Atcor, Australia. |
| Skin autofluorescence | Advanced end glycation products (AGEs) | AGEs Reader, DiagnOptics, Groningen, The Netherlands |

| | | |
|---|---|---|
| Endothelial function | Reactive Hyperemia Index (RHI) | Flow-mediated dilation, EndoPat, Itamar, Israel |

**Supplemental Table S2. Age-dependence of kidney function estimates on AGE level in men and women. Interaction analysis.**

| MEN | | | | | | |
|---|---|---|---|---|---|---|
| Model | Unstandardized Coefficients | | P-value | Correlations | | |
| | B | SE | | Zero-order | Partial | Part |
| Age (centered) | 0.236 | 0.014 | <.001 | 0.405 | 0.355 | 0.347 |
| eGFRaverage (centered) | 0.003 | 0.015 | 0.813 | -0.207 | 0.005 | 0.005 |
| Interaction: Age*eGFRaverage (both centered) | 0.001 | 0.013 | 0.969 | -0.042 | 0.001 | 0.001 |
| | | | | | | |
| Age (centered) | 0.229 | 0.013 | <.001 | 0.405 | 0.359 | 0.352 |
| eGFRcys (centered) | -0.006 | 0.014 | 0.673 | -0.189 | -0.01 | -0.009 |
| Interaction: Age*eGFRcys (both centered) | -0.024 | 0.014 | 0.088 | -0.086 | -0.039 | -0.035 |
| | | | | | | |
| Age (centered) | 0.242 | 0.013 | <.001 | 0.405 | 0.384 | 0.379 |
| eGFRcr (centered) | 0.017 | 0.013 | 0.187 | -0.151 | 0.03 | 0.027 |
| Interaction: Age*eGFRcr (both centered) | 0.033 | 0.012 | 0.006 | 0.042 | 0.062 | 0.056 |
| | | | | | | |
| Age (centered) | 0.226 | 0.012 | <.001 | 0.405 | 0.388 | 0.383 |
| eGFRcys/eGFRcr (centered) | -0.018 | 0.013 | 0.16 | -0.097 | -0.032 | -0.029 |
| Interaction: Age*eGFRcys/eGFRcr (both centered) | -0.046 | 0.014 | <.001 | -0.113 | -0.077 | -0.07 |

| WOMEN | | | | | | |
|---|---|---|---|---|---|---|
| Model | Unstandardized Coefficients | | P-value | Correlations | | |
| | B | SE | | Zero-order | Partial | Part |
| Age (centered) | 0.288 | 0.012 | <.001 | 0.517 | 0.463 | 0.447 |
| eGFRaverage (centered) | 0.026 | 0.012 | 0.028 | -0.257 | 0.049 | 0.042 |
| Interaction: Age*eGFRaverage (both centered) | -0.017 | 0.011 | 0.113 | -0.049 | -0.035 | -0.03 |
| | | | | | | |
| Age (centered) | 0.287 | 0.012 | <.001 | 0.517 | 0.484 | 0.472 |
| eGFRcys (centered) | 0.027 | 0.011 | 0.015 | -0.202 | 0.054 | 0.046 |
| Interaction: Age*eGFRcys (both centered) | -0.032 | 0.01 | 0.002 | -0.069 | -0.068 | -0.058 |
| | | | | | | |

| | | | | | | |
|---|---|---|---|---|---|---|
| Age (centered) | 0.275 | 0.011 | <.001 | 0.517 | 0.473 | 0.459 |
| eGFRcr (centered) | 0.001 | 0.011 | 0.959 | -0.24 | 0.001 | 0.001 |
| Interaction: Age*eGFRcr (both centered) | 0.025 | 0.011 | 0.024 | 0.013 | 0.05 | 0.043 |
| | | | | | | |
| Age (centered) | 0.279 | 0.01 | <.001 | 0.517 | 0.517 | 0.514 |
| eGFRcys/eGFRcr (centered) | 0.02 | 0.01 | 0.037 | -0.063 | 0.047 | 0.04 |
| Interaction: Age*eGFRcys/eGFRcr (both centered) | -0.041 | 0.01 | <.001 | -0.076 | -0.093 | -0.08 |

The results of interaction analysis to examine whether the relationship between kidney function estimates and AGE level is age-dependent. Linear regression models where AGEs is dependent variable.

Abbreviations: AGEs, advanced glycation end products; eGFR, estimated glomerular filtration rate mL/min/1.73m2; eGFRcys, cystatin C-based eGFR; eGFRcr, creatinine-based eGFR; eGFRaverage, average eGFR; eGFRcys/eGFRcr, ratio between cystatin C eGFR and creatinine-based eGFR;

**Supplemental Table S3. Effect size of eGFRcys/eGFRcr on AGEs across different age groups. Linear regression analysis.**

| **MEN** | | | | | | | |
|---|---|---|---|---|---|---|---|
| Age group | Model | Unstandardized Coefficients | | P-value | Correlations | | |
| | | B | SE | | Zero-order | Partial | Part |
| <30 | eGFRcys/eGFRcr | 0.281 | 0.141 | 0.047 | 0.085 | 0.085 | 0.085 |
| 30-50 | eGFRcys/eGFRcr | -0.215 | 0.077 | 0.006 | -0.105 | -0.105 | -0.105 |
| >50 | eGFRcys/eGFRcr | -0.27 | 0.094 | 0.004 | -0.108 | -0.108 | -0.108 |
| **WOMEN** | | | | | | | |
| Age group | Model | Unstandardized Coefficients | | P-value | Correlations | | |
| | | B | SE | | Zero-order | Partial | Part |
| <30 | eGFRcys/eGFRcr | 0.258 | 0.06 | <0.001 | 0.178 | 0.178 | 0.178 |
| 30-50 | eGFRcys/eGFRcr | 0.199 | 0.064 | 0.002 | 0.118 | 0.118 | 0.118 |
| >50 | eGFRcys/eGFRcr | -0.25 | 0.097 | 0.01 | -0.094 | -0.094 | -0.094 |

Linear regression models where AGEs is dependent variable.

Abbreviations: AGEs, advanced glycation end products; eGFR, estimated glomerular filtration rate mL/min/1.73m2; eGFRcys, cystatin C-based eGFR; eGFRcr, creatinine-based eGFR; eGFRcys/eGFRcr, ratio between cystatin C eGFR and creatinine-based eGFR;

**Supplemental Table S~~2~~4. Comparison of four pathophysiological patterns when combining advanced end-glycation product and endothelial function.**

| | AGEs <1.6 + EF | | | AGEs<1.6 + ED | | | AGEs≥1.6 + EF | | | AGEs≥1.6 + ED | | | In men | In women |
|---|---|---|---|---|---|---|---|---|---|---|---|---|---|---|
| | **Women** | **Men** | **P†** | **Women** | **Men** | **P†** | **Women** | **Men** | **P†** | **Women** | **Men** | **P†** | **P ‡** | **P ‡** |
| | **N=268** | **N=234** | | **N=129** | **N=121** | | **N=519** | **N=517** | | **N=220** | **N=200** | | | |
| Age, years | 46(11) | 47(11) | 0.338 | 40(13) | 42(13) | 0.343 | 53(7) | 54(8) | 0.458 | 53(9) | 52(9) | 0.58 | <0.001 | <0.001 |
| Waist, cm | 91(13) | 89(11) | 0.031 | 89(13) | 92(11) | 0.069 | 92(14) | 94(13) | 0.046 | 94(14) | 97(13) | 0.032 | <0.001 | 0.011 |
| BMI | 26.1(4.4) | 25.7(4.2) | 0.379 | 25.6(4.3) | 26.5(4.0) | 0.379 | 26.4(4.7) | 27.2(5.0) | 0.015 | 27.1(4.7) | 27.5(4.2) | 0.456 | <0.001 | 0.01 |
| Hypertension, yes | 22(59) | 18(41) | 0.082 | 13(17) | 17(20) | 0.454 | 25(130) | 29(150) | 0.159 | 26(58) | 26(51) | 0.899 | <0.001 | 0.02 |
| Antihypertensive treatment, yes | 10(28) | 8(19) | 0.238 | 5(7) | 10(12) | 0.179 | 14(70) | 17(87) | 0.133 | 18(39) | 15(30) | 0.465 | 0.004 | 0.005 |
| History of CVD, yes | 0.7(2) | 0.9(2) | 0.924 | 0(0) | 3(4) | 0.036 | 3(14) | 4(19) | 0.389 | 6(13) | 4(7) | 0.265 | 0.171 | <0.001 |
| Smoking, yes | 8(22) | 8(18) | 0.407 | 12(16) | 10(12) | 0.543 | 10(54) | 10(47) | 0.098 | 14(30) | 15(30) | 0.777 | <0.001 | <0.001 |
| **Biochemistry** | | | | | | | | | | | | | | |
| Fasting glycemia | 5.3(0.6) | 5.2(0.6) | 0.208 | 5.2(0.5) | 5.3(0.5) | 0.171 | 5.4(0.6) | 5.3(0.6) | 0.469 | 5.3(0.6) | 5.4(0.6) | 0.444 | 0.039 | 0.046 |
| TG | 0.9(0.8) | 0.8(0.8) | 0.158 | 1.0(0.7) | 0.9(0.8) | 0.638 | 0.9(0.7) | 1.0(0.8) | 0.498 | 0.9(0.7) | 1.2(0.9) | 0.026 | <0.001 | 0.564 |
| HDL | 1.6(0.4) | 1.7(0.5) | 0.102 | 1.6(0.5) | 1.6(0.5) | 0.246 | 1.7(0.5) | 1.6(0.5) | <0.001 | 1.7(0.5) | 1.6(0.5) | 0.032 | 0.009 | 0.004 |
| LDL | 3.3(1.0) | 3.4(0.9) | 0.33 | 3.0(1.0) | 3.1(0.9) | 0.332 | 3.5(1.0) | 3.5(0.9) | 0.135 | 3.4(1.0) | 3.5(1.0) | 0.217 | <0.001 | <0.001 |
| HbA1c | 34(3) | 34(3) | 0.399 | 34(4) | 35(4) | 0.437 | 35(4) | 36(4) | 0.782 | 36(4) | 35(3) | 0.307 | <0.001 | <0.001 |
| **Kidney function** | | | | | | | | | | | | | | |
| eGFRcys | 92(22) | 90(18) | 0.243 | 99(25) | 97(19) | 0.344 | 85(17) | 84(18) | 0.595 | 87(21) | 85(17) | 0.41 | <0.001 | <0.001 |
| eGRcr | 80(10) | 80(10) | 0.956 | 80(10) | 80(10) | 0.953 | 78(9) | 79(19) | 0.044 | 80(10) | 81(9) | 0.088 | 0.108 | 0.006 |
| eGFRaverage | 86(13) | 85(12) | 0.357 | 90(14) | 88(12) | 0.399 | 81(11) | 82(12) | 0.662 | 83(13) | 83(11) | 0.989 | <0.001 | <0.001 |
| eGFRcys/eGFRcr | 1.2(0.3) | 1.1(0.2) | 0.231 | 1.3(0.3) | 1.2(0.3) | 0.368 | 1.1(0.2) | 1.1(0.2) | 0.072 | 1.1(0.3) | 1.1(0.2) | 0.065 | <0.001 | <0.001 |
| log(UACR) | -0.77(0.92) | -0.74(0.90) | 0.276 | -1.04(0.67) | -1.01(1.16) | 0.93 | -0.67(0.81) | -0.65(1.02) | 0.752 | -0.62(1.00) | -0.63(0.90) | 0.969 | 0.018 | 0.777 |

| | | | | | | | | | | | | | |
|---|---|---|---|---|---|---|---|---|---|---|---|---|---|
| UACR >3.0 mg/mmoL | 1(3) | 3(6) | 0.246 | 0(0) | 2(2) | 0.134 | 2(7) | 2(10) | 0.439 | 4(6) | 2(3) | 0.347 | 0.907 | 0.176 |
| **Vascular parameters** | | | | | | | | | | | | | | |
| SBP | 118(16) | 119(15) | 0.702 | 113(12) | 117(12) | 0.01 | 122(17) | 123(17) | 0.407 | 118(14) | 119(13) | 0.432 | <0.001 | <0.001 |
| DBP | 74(10) | 74(10) | 0.655 | 71(8) | 71(8) | 0.639 | 77(10) | 77(10) | 0.503 | 74(9) | 75(8) | 0.782 | <0.001 | <0.001 |
| HR | 60(9) | 61(9) | 0.175 | 61(9) | 59(8) | 0.187 | 61(9) | 61(9) | 0.701 | 61(9) | 61(9) | 0.65 | 0.227 | 0.469 |
| **Skin autofluorescence** | | | | | | | | | | | | | | |
| AGE (AU) | 1.27(0.25) | 1.18(0.24) | 0.051 | 1.26(0.25) | 1.26(0.25) | 0.722 | 2.08(0.34) | 2.05(0.27) | 0.073 | 2.09(0.35) | 2.04(0.34) | 0.087 | <0.001 | <0.001 |
| **Flow-mediated dilation** | | | | | | | | | | | | | | |
| RHI | 2.4(0.6) | 2.4(0.5) | 0.805 | 1.4(0.2) | 1.4(0.2) | 0.887 | 2.4(0.5) | 2.3(0.5) | 0.295 | 1.4(0.2) | 1.4(0.2) | 0.995 | <0.001 | <0.001 |

† P-values for sex differences within the group

‡ Sex-specific P-values for differences in variables among the groups

Abbreviations: BMI, body mass index kg/m2; TG, triglycerides mmoL/L; HDL, high-density lipoproteins, mmoL/L; LDL, low-density lipoproteins mmoL/L; HbA1c, glycolized hemoglobin IFCC units; eGFR, estimated glomerular filtration rate mL/min/1.73m2; eGFRcys, cystatin C-based eGFR; eGFRcr, creatinine-based eGFR; eGFRaverage, average eGFR; eGFRcys/eGFRcr, ratio between cystatin C eGFR and creatinine-based eGFR; UACR, urine albumin-to-creatinine ratio mg/mmoL; log(UACR), natural algorithm of uACR; SBP, systolic blood pressure mmHg; DBP, diastolic blood pressure mmHg; HR, heart rate bpm; AGE, advanced glycation end-products AU; RHI, reactive hyperemia index.

UACR is available in 866 men and 911 women

**Supplemental Figure 1 Sex-specific correlation heatmaps**

Women

| | eGFRcys | eGFRcr | eGFRav | log(UACR) | AGEs | log(RHI) | Age | BMI | Waist | SBP | DBP | Glycemia | HbA1c | TG | HDL | LDL |
|---|---|---|---|---|---|---|---|---|---|---|---|---|---|---|---|---|
| eGFRcys/eGFRcr | 0.829 | -0.339 | 0.389 | -0.024 | -0.044 | -0.069 | -0.189 | -0.303 | -0.393 | -0.157 | -0.182 | 0.005 | -0.107 | -0.086 | 0.262 | -0.163 |
| eGFRcys | | 0.231 | 0.722 | -0.033 | -0.191 | -0.087 | -0.413 | -0.302 | -0.382 | -0.278 | -0.281 | -0.022 | -0.206 | -0.131 | 0.183 | -0.266 |
| eGFRcr | | | 0.588 | -0.021 | -0.261 | -0.040 | -0.382 | 0.025 | 0.049 | -0.199 | -0.165 | -0.047 | -0.167 | -0.071 | -0.166 | -0.178 |
| eGFRav | | | | -0.097 | -0.304 | -0.060 | -0.517 | -0.229 | -0.280 | -0.306 | -0.307 | -0.043 | -0.237 | -0.166 | 0.033 | -0.291 |
| log(uACR) | | | | | 0.249 | 0.009 | 0.119 | 0.036 | -0.018 | 0.108 | 0.135 | 0.113 | 0.107 | 0.182 | 0.068 | 0.002 |
| AGEs | | | | | | 0.001 | 0.541 | 0.076 | 0.053 | 0.197 | 0.279 | 0.168 | 0.241 | 0.148 | 0.205 | 0.178 |
| log(RHI) | | | | | | | 0.157 | -0.065 | -0.047 | 0.161 | 0.140 | 0.032 | -0.024 | -0.112 | 0.085 | 0.062 |
| Age | | | | | | | | 0.244 | 0.345 | 0.471 | 0.534 | 0.214 | 0.374 | 0.150 | 0.155 | 0.425 |
| BMI | | | | | | | | | 0.87 | 0.316 | 0.368 | 0.098 | 0.191 | 0.319 | -0.351 | 0.263 |
| Waist | | | | | | | | | | 0.394 | 0.400 | 0.068 | 0.233 | 0.283 | -0.413 | 0.335 |
| SBP | | | | | | | | | | | 0.799 | 0.125 | 0.202 | 0.145 | -0.053 | 0.293 |
| DBP | | | | | | | | | | | | 0.134 | 0.214 | 0.265 | -0.004 | 0.353 |
| Glycemia | | | | | | | | | | | | | 0.274 | -0.023 | 0.014 | -0.128 |
| HbA1c | | | | | | | | | | | | | | 0.176 | -0.034 | 0.263 |
| TG | | | | | | | | | | | | | | | -0.222 | 0.382 |
| HDL | | | | | | | | | | | | | | | | -0.084 |
| LDL | | | | | | | | | | | | | | | | |

Men

| | eGFRcys | eGFRcr | eGFRav | log(UACR) | AGEs | log(RHI) | Age | BMI | Waist | SBP | DBP | Glycemia | HbA1c | TG | HDL | LDL |
|---|---|---|---|---|---|---|---|---|---|---|---|---|---|---|---|---|
| eGFRcys/eGFRcr | 0.775 | -0.266 | 0.350 | -0.106 | -0.098 | -0.075 | -0.189 | -0.189 | -0.162 | 0.000 | -0.169 | -0.018 | -0.135 | -0.099 | 0.005 | -0.079 |
| eGFRcys | | 0.388 | 0.778 | -0.097 | -0.203 | -0.098 | -0.454 | -0.254 | -0.263 | -0.206 | -0.315 | -0.082 | -0.310 | -0.173 | -0.023 | -0.225 |
| eGFRcr | | | 0.709 | 0.007 | -0.178 | -0.049 | -0.433 | -0.115 | -0.177 | -0.325 | -0.248 | -0.102 | -0.259 | -0.128 | -0.041 | -0.241 |
| eGFRav | | | | -0.126 | -0.248 | -0.102 | -0.547 | -0.236 | -0.278 | -0.306 | -0.357 | -0.092 | -0.328 | -0.191 | -0.018 | -0.281 |
| log(uACR) | | | | | 0.067 | 0.046 | 0.176 | 0.057 | 0.032 | 0.121 | 0.188 | 0.012 | 0.032 | -0.062 | 0.078 | 0.046 |
| AGEs | | | | | | 0.017 | 0.400 | 0.172 | 0.280 | 0.236 | 0.243 | 0.114 | 0.221 | 0.180 | -0.049 | 0.209 |
| log(RHI) | | | | | | | 0.142 | -0.110 | -0.152 | 0.164 | 0.187 | -0.050 | -0.045 | -0.104 | 0.125 | 0.040 |
| Age | | | | | | | | 0.236 | 0.332 | 0.395 | 0.538 | 0.17 | 0.435 | 0.094 | 0.208 | 0.398 |
| BMI | | | | | | | | | 0.855 | 0.285 | 0.341 | 0.134 | 0.218 | 0.34 | -0.302 | 0.255 |
| Waist | | | | | | | | | | 0.422 | 0.406 | 0.217 | 0.276 | 0.49 | -0.380 | 0.337 |
| SBP | | | | | | | | | | | 0.763 | 0.206 | 0.249 | 0.309 | -0.067 | 0.287 |
| DBP | | | | | | | | | | | | 0.118 | 0.251 | 0.235 | 0.014 | 0.362 |
| Glycemia | | | | | | | | | | | | | 0.285 | 0.067 | -0.125 | -0.099 |
| HbA1c | | | | | | | | | | | | | | 0.141 | -0.01 | 0.260 |
| TG | | | | | | | | | | | | | | | -0.457 | 0.368 |
| HDL | | | | | | | | | | | | | | | | -0.074 |
| LDL | | | | | | | | | | | | | | | | |

Suppl Fig 1 depicts a sex-disaggregated heatmap for Pearson correlation coefficients. In women, lower eGFRcys/eGFRcr ratio was correlated with higher age, increased body mass index (BMI) and waist circumference, elevated SBP and DBP, higher LDL, higher eGFRcr and very weakly with higher HbA1c, RHI and TG. Additionally, a lower eGFRcys/eGFRcr ratio was associated with lower average eGFR, lower eGFRcys and lower HDL levels. AGEs were not linearly correlated to eGFRcys/eGFRcr ratio. In men, the correlations of eGFRcys/eGFRcr ratio with other variables were similar, except for a weak negative correlation with UACR and AGEs, and no significant correlation with HDL and SBP. AGEs were negatively correlated with eGFRcys, eGFRcr and average eGFR in both sexes and positively correlated to UACR only in women. Abbreviations: BMI, body mass index kg/$m^2$; TG, triglycerides mmoL/L; HDL, high-density lipoproteins, mmoL/L; LDL, low-density lipoproteins mmoL/L; HbA1c, glycolized haemoglobin IFCC units; eGFR, estimated glomerular filtration rate mL/min/1.73$m^2$; eGFRcys, cystatin C-based eGFR; eGFRcr, creatinine-based eGFR; eGFRaverage, average eGFR; eGFRcys/eGFRcr, ratio between cystatin C

eGFR and creatinine-based eGFR; log(UACR), logarithm of urine albumin-to-creatinine ratio mg/mmoL; SBP, systolic blood pressure mmHg; DBP, diastolic blood pressure mmHg; AGE, advanced glycation end-products AU; log(RHI), logarithm of reactive hyperemia index.

**Supplemental Figure 2 Linear regression coefficients for Model 4 where AGEs are a dependent factor. Forrest plot diagram.**

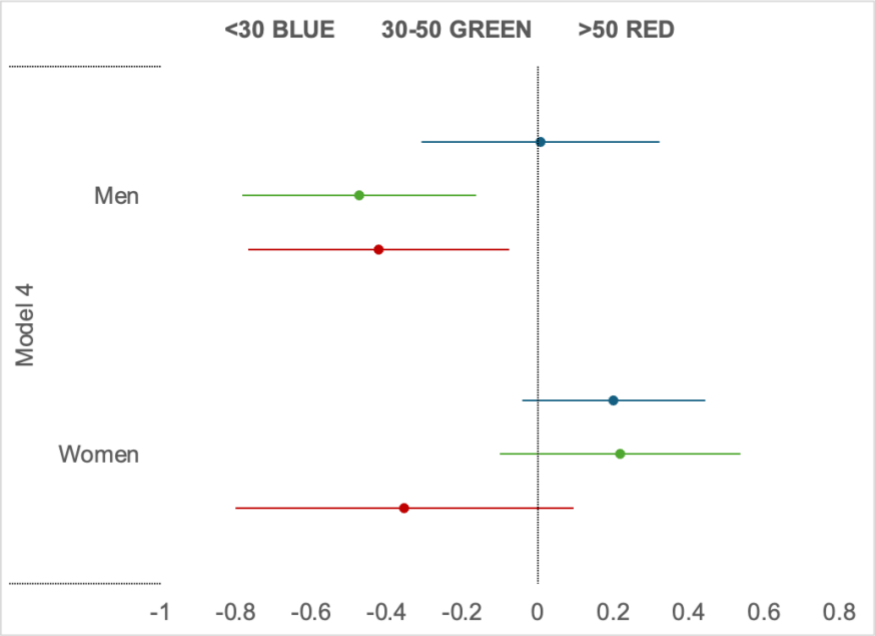


Suppl Fig 2 depicts sex and age group disaggregated linear regression analysis. Forrest plot for beta coefficients (round dots) and their 95%CI. Blue – ages under 30; green – from 30 to 50; red – above 50.

Model 4: eGFRcys/eGFRcr adjusted for eGFRaverage, age, body mass index, smoking, fasting plasma glucose, triglycerides, LDL-cholesterol, HDL-cholesterol, HbA1c, systolic blood pressure, antihypertensive treatment and log (UACR).
